# Beyond the cognitive haze: BrainRot, cognitive flexibility, physical exercise, and anxiety symptoms among Chinese adolescents

**DOI:** 10.3389/fpsyt.2026.1834197

**Published:** 2026-08-27

**Authors:** Yuhan Chen, Zhijia Zhang, Chuqi Yan, Chenyang Xu, Zhen Guo, Zhiru Liang, Jing Wang, Yang Liu

**Affiliations:** 1 Jishou University, Jishou, China; 2 Finance Department, Xilingol League Central Hospital, Xilin Hot, Inner Mongolia, China; 3 Institute of Reproductive Medicine, Medical School, Nantong University, Nantong, China; 4 Department of Hepatobiliary Surgery, Xilingol League Central Hospital, Xilin Hot, Inner Mongolia, China; 5 Department of Anesthesiology for Surgery, Xilingol League Central Hospital, Xilin Hot, Inner Mongolia, China; 6 Chengdu Experimental Middle School, Chengdu, China

**Keywords:** adolescents, anxiety symptoms, BrainRot, cognitive flexibility, physical exercise

## Abstract

**Introduction:**

The present study examined BrainRot, cognitive flexibility, anxiety symptoms, and physical exercise in Chinese adolescents.

**Methods:**

A cross-sectional design was employed, with data collected from 3,261 adolescents through questionnaires assessing problematic short-video use, cognitive flexibility, anxiety symptoms, and physical exercise.

**Results:**

BrainRot was positively related to anxiety symptoms (r = 0.329) and negatively related to cognitive flexibility (r = −0.286) and physical exercise (r = −0.105). Anxiety symptoms were also negatively related to cognitive flexibility (r = −0.357) and physical exercise (r = −0.114), whereas physical exercise was positively related to cognitive flexibility (r = 0.123). Mediation analysis suggested that cognitive flexibility may statistically mediate the link between BrainRot and anxiety symptoms. Furthermore, physical exercise moderated the path from BrainRot to cognitive flexibility and the direct path from BrainRot to anxiety symptoms, showing pathway-specific patterns. Conditional indirect effects through cognitive flexibility were observed at low, mean, and high levels of physical exercise, suggesting that physical exercise may play a pathway-specific role in shaping the associations among BrainRot, cognitive flexibility, and anxiety symptoms.

**Discussion:**

These findings deepen our understanding of the pathway-specific role of physical exercise in the associations among BrainRot, cognitive flexibility, and anxiety symptoms, and may inform future strategies for adolescent mental health promotion.

## 1 Introduction

BrainRot is a term used to describe the mental and intellectual deterioration resulting from excessive consumption of low-quality online content. As defined in the Oxford Dictionary, BrainRot refers to a perceived decline in mental or intellectual functioning caused by prolonged exposure to low-quality online content. In this study, BrainRot refers to adolescents’ problematic short-video use rather than a formal clinical diagnosis. Short-video platforms such as TikTok and Douyin use algorithmic recommendation, fragmented content delivery, instant gratification mechanisms, and intense visual stimuli to sustain user engagement. These features may promote prolonged immersion, distract attention, and weaken information processing, thereby making BrainRot a vivid description of the negative cognitive consequences associated with problematic short-video use (1, 2). As a world-renowned short-form video sharing service, TikTok has more than 1.677 billion registered users and approximately 1.1 billion active users across more than 160 countries and regions (3). According to the 2024 China Internet Network Information Center report, the number of short-video users in China reached 1.05 billion. Constituting exactly 95.5% of the comprehensive online population, this vast consumer base highlights a continuously expanding adolescent demographic (4). The excessive use of short video platforms has often been criticized for diminishing people’s attention span and cognitive depth, potentially leading to issues such as student depression (5), instant gratification dependence (6), social anxiety (7), vision impairment (8), and sleep disturbances (9). According to Uses and Gratifications (U&G) theory (10), BrainRot arises because platforms effectively meet users’ diverse needs, including information, entertainment, social interaction, identity, and emotional support. Platform-based instant rewards and social interaction may further reinforce repeated use, which may develop into problematic short-video use for some adolescents (11). Therefore, studying adolescent BrainRot is crucial. In the digital age, the phenomenon of BrainRot has become increasingly prominent. A growing body of research suggests that excessive use of short videos not only impacts individuals’ cognitive abilities but is also closely related to mental health issues (12). Among these, anxiety, as an emotional response, typically manifests as concern about uncertainty and potential threats related to future events (13). The onset and maintenance of anxiety, beyond exerting far-reaching impacts on the mental health of each individual, shape critical functional domains including behavioral decision-making, emotional regulation, alongside lifestyle choices (14). A 2021 meta-analysis, incorporating 29 studies, identified a 20.5% global prevalence of anxiety symptoms within adolescent populations (15). Another meta-analysis conducted in 2022, focusing on anxiety symptoms during the COVID-19 pandemic, involved 25 studies with a total of 1,003,743 Chinese adolescents. The results revealed that the overall prevalence of anxiety symptoms was 25.0% (16). Consequently, clarifying how BrainRot relates to adolescent mental health has become an important issue. According to emotional regulation theory, individuals manage and regulate their emotions in various ways. Healthy emotional regulation helps individuals adapt to external environments, while unhealthy emotional regulation strategies (such as avoidance, suppression, or over-reliance on certain behaviors) may lead to psychological problems (17, 18). Excessive dependence on short videos may become an unhealthy emotional regulation strategy, particularly when individuals are faced with emotional distress such as anxiety, loneliness, or stress. Adolescents may use short videos as a means of escaping negative emotions; however, the short-term emotional relief provided by this behavior is insufficient to resolve deeper emotional issues. Long-term reliance on this instant gratification mechanism may lead to a deterioration in emotional regulation abilities, increasing the risk of anxiety (19, 20). Therefore, BrainRot may be considered a relevant correlate of anxiety symptoms. Empirical evidence has linked problematic short-video use to higher anxiety symptoms among adolescents. For example, a systematic review on adolescent social media addiction and mental health, which included 32 studies, reported that problematic short-video use was significantly associated with higher anxiety symptoms among adolescents. Greater cognitive flexibility corresponded to fewer anxiety symptoms (21). In summary, this study hypothesizes that there is a positive correlation between BrainRot and adolescent anxiety symptoms (H1).

Executive function refers to a set of high-level cognitive processes that help individuals plan, manage, and regulate behavior in complex environments to achieve goals (22). Cognitive flexibility is considered a key component of executive function. It refers to an individual’s ability to adjust cognitive patterns across different contexts, including switching thoughts, adjusting cognitive strategies, and adapting to new environments (23). A recent multinational study of 378,087 adolescents from 57 countries reported a close link between cognitive flexibility and adolescents’ academic performance (24). Another study indicated that cognitive flexibility varies across an individual’s lifespan (25). Therefore, enhancing cognitive flexibility in adolescents is crucial for promoting their physical and mental health development. According to the attentional resource theory, individuals have a limited amount of attention, and prolonged focus on a single stimulus may constrain other cognitive functions (26). The high frequency, short duration, and immediate feedback characteristics of short videos may lead to excessive depletion of adolescents’ attentional resources, consequently affecting other essential cognitive functions and reducing cognitive flexibility. In other words, adolescents experiencing BrainRot may have a diminished ability to cope with complex tasks or adapt to environmental changes, potentially becoming trapped in mechanized thought and behavior patterns (27). Empirical evidence has also reported an inverse association between problematic short-video use and cognitive flexibility. For example, a cross-sectional study of 368 Chinese adolescents reported that higher BrainRot scores corresponded to poorer cognitive function (28). Furthermore, cognitive flexibility is also associated with adolescent mental health. According to cognitive-behavioral theory (29), the core of anxiety lies in negative cognitive biases, where individuals interpret themselves, others, and the world through fixed negative thought patterns. These rigid thinking styles exacerbate the formation and maintenance of anxiety. Individuals with lower cognitive flexibility are more likely to persist in negative thinking, such as catastrophizing or overgeneralizing, which intensifies anxiety symptoms (30). The development of anxiety symptoms is closely related to an individual’s lack of flexible thinking when faced with challenges. Those with limited cognitive flexibility may struggle to effectively alter negative thoughts in difficult situations, leading to feelings of sadness and helplessness. In contrast, individuals with higher cognitive flexibility can alleviate emotional distress by changing their thought processes and reassessing the situation (31, 32). Earlier evidence has linked cognitive flexibility to adolescent anxiety symptoms. Impaired executive function may lead to difficulties in emotional regulation, increasing the accumulation of negative emotions. For instance, an experimental study found that high levels of executive function were associated with effective emotional regulation strategies, and the content of executive function tasks did not affect the relationship between executive function and emotional regulation (33). These findings suggest that cognitive flexibility may be closely related to adolescent anxiety symptoms. In conclusion, this study hypothesizes that cognitive flexibility mediates the relationship between BrainRot and anxiety symptoms (H2).

Physical exercise has been widely recognized as an important behavior for promoting physical and mental health. In the present study, it refers to planned and regular bodily activity that may contribute to better psychological well-being among adolescents (34). Self-determination theory emphasizes intrinsic motivation and autonomy in behavioral activities (35). According to this theory, when individuals’ basic psychological needs (such as autonomy, competence, and relatedness) are satisfied, they are more likely to exhibit positive behaviors and maintain a healthy psychological state. Exercise is considered to facilitate the fulfillment of these basic needs. In the context of BrainRot, individuals may become trapped in the condition due to a lack of autonomy, control, and a sense of belonging. However, physical exercise can provide an autonomous behavioral choice and improve self-determination by enhancing competence (through physical skills), relatedness (via social connections in team sports), and self-control (through regulating physical states and emotions). Therefore, physical exercise may be associated with better emotional regulation and cognitive functioning among adolescents (36). Prior research has indicated that physical exercise has a moderating effect on both BrainRot and cognitive flexibility. For example, a cross-sectional study involving 756 Chinese adolescents found that adolescents reporting more physical exercise tended to show lower levels of BrainRot (37). Another cross-sectional study involving 500 adolescents found that adolescents with higher physical exercise levels tended to report better cognitive flexibility (38). Additionally, according to the neurobiological model, individuals’ behavior, emotions, and cognitive functions are closely linked to the structure of the brain and neurotransmitter systems (39). From a neurobiological perspective, adolescents’ behavioral, emotional, and cognitive functioning may be closely related to brain systems and neurotransmitter activity (40). Although the present study did not directly examine neural or biological indicators, prior research has suggested that repeated engagement with fast-paced and highly rewarding short-video content may be linked to increased reward sensitivity and reduced cognitive control. This may provide a possible explanation for the association between BrainRot and anxiety symptoms among adolescents. The immediate rewards provided by short videos may reduce interest in real-world activities and contribute to emotional distress. Physical exercise has been linked to neurotransmitter regulation, emotional adjustment, and cognitive functioning, which may provide a possible explanation for its association with better mental health outcomes. Physical exercise has been associated with better executive control, self-regulation, and mental health outcomes, which may partly explain its potential role in weakening the association between BrainRot and anxiety symptoms (41). This pattern is further supported by a recent systematic review and best-evidence synthesis of 247 studies on physical activity and mental health, including 173 mediation studies and 82 moderation studies. The review highlighted multiple psychological, social, behavioral, physiological, neurobiological, and cognitive pathways linking physical activity to mental health outcomes (42). Therefore, physical exercise may help explain individual differences in the association between BrainRot and anxiety symptoms. In conclusion, this study hypothesizes that physical exercise moderates the relationships between BrainRot and cognitive flexibility, as well as between BrainRot and anxiety symptoms (H3).

**Figure 1:**
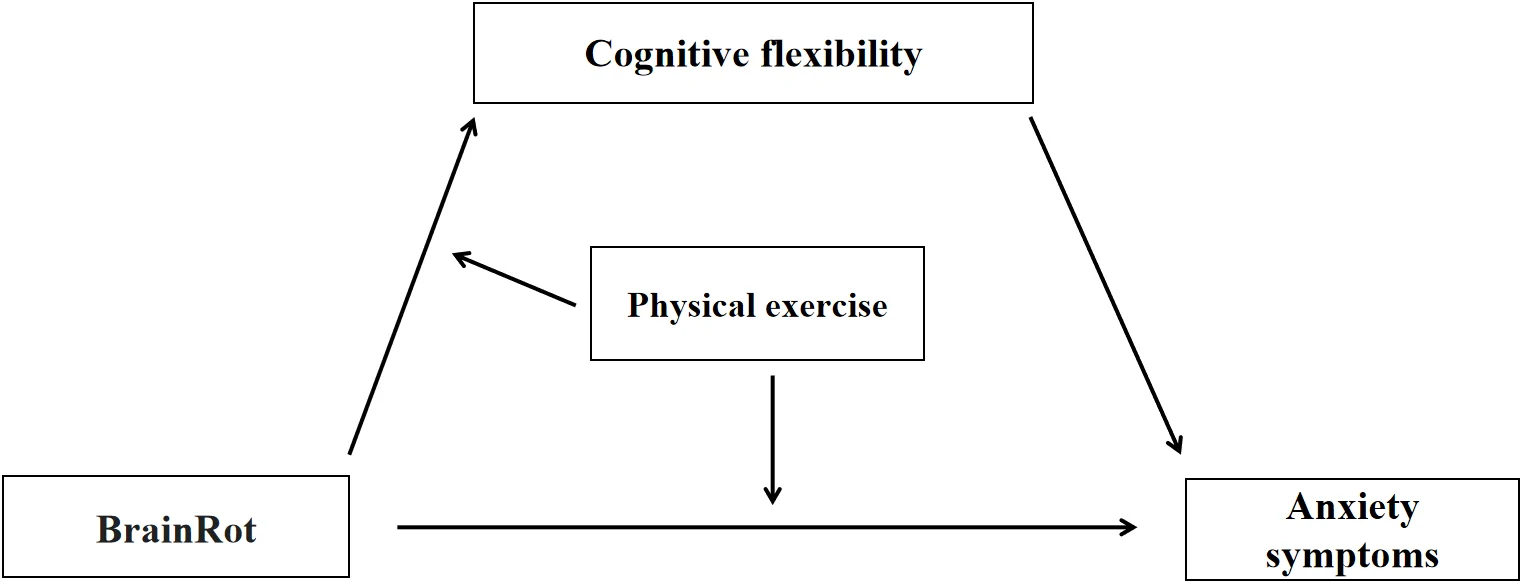
Hypothesized moderated mediation model.

Drawing on the aforementioned evidence, BrainRot was expected to be positively associated with anxiety symptoms, and cognitive flexibility was proposed as a potential mediator in this association. Additionally, physical exercise moderates the relationships between BrainRot and cognitive flexibility, as well as between BrainRot and anxiety. This study aims to construct the following moderated mediation model (see **Figure 1**).

## 2 Methods

### 2.1 Participants

Ethical approval was obtained from the Ethics Committee of Jishou University on November 1, 2025, before the initiation of formal data collection. In November 2025, after ethics approval had been granted, adolescents were recruited from six elementary, middle, and high schools in Anhui, Shanxi, Shaanxi, Sichuan, and Hunan Provinces using convenience sampling. Web-based questionnaires were distributed by class teachers through parent group chats. Parents or legal guardians first reviewed the study information sheet and informed consent statement. After parental or guardian consent was obtained, adolescents completed the questionnaire online. Adolescent assent was also obtained on the first page of the online questionnaire. The opening page explained the study purpose, anonymity, confidentiality, voluntary participation, and the right to withdraw at any time without penalty. Only adolescents who selected “Agree” could proceed to the formal questionnaire. Participants completed the questionnaire in less than 10 minutes. Data screening was conducted before statistical analysis. A total of 3,375 questionnaires were initially returned. After screening, 114 questionnaires were excluded because of incomplete responses, unusually short completion times, patterned responding, or obvious logical inconsistencies. Finally, 3,261 valid questionnaires were retained for analysis, yielding a valid response rate of 96.6%. The final sample included 1,622 male participants (49.7%) and 1,639 female participants (50.3%). The average age was 14.83 ± 1.40 years. Regarding hukou status, which refers to China’s household registration system, 1,590 participants (48.8%) had rural household registration, and 1,671 participants (51.2%) had urban household registration. There were 146 elementary school students (4.5%), 1,269 middle school students (38.9%), and 1,846 high school students (56.6%). Additionally, 2,149 participants (65.9%) were boarding students, while 1,112 (34.1%) were day students. Regarding family structure, 380 participants (11.7%) were only children, and 2,881 (88.3%) were non-only children.

### 2.2 Measurement tools

#### 2.2.1 BrainRot

BrainRot was operationally defined as short-video addiction and was measured using the Short Video Addiction Scale developed by Mao and Jiang (43), with reference to its subsequent application by Mao Zheng (44). This scale assesses adolescents’ problematic short-video use rather than a formal clinical diagnosis. It consists of 13 items across three dimensions: cognitive-behavioral changes, physiological impairment, and social attachment. Each item is rated on a 5-point Likert scale ranging from 1 (strongly disagree) to 5 (strongly agree), with higher total scores indicating more severe problematic short-video use. In the present study, the scale showed good internal consistency, with a Cronbach’s alpha coefficient of 0.870.

#### 2.2.2 Cognitive flexibility

For the current investigation, cognitive flexibility was gauged with the Executive Function Scale, an instrument compiled by Huang Chunhui and fellow researchers (45), with the cognitive flexibility subscale selected for formal assessment. This subscale encompasses six measurement items in total. A 3-point Likert rating system was adopted for response scoring, with responses spanning 1 (often) to 3 (never). In the present study, the cognitive flexibility items were reverse-coded so that higher scores indicated fewer difficulties and better cognitive flexibility. The total score was calculated by summing the reverse-coded items. For the sample recruited in this work, the Cronbach’s alpha coefficient of this measurement tool reached 0.858.

#### 2.2.3 Anxiety symptoms

To evaluate anxiety symptoms among adolescents, the present study used the GAD-2 (46). Although the GAD-2 is conventionally scored from 0 to 3 per item, the four response options in the present online questionnaire were coded from 1 to 4 for data-entry convenience. This coding is equivalent to adding one point to each item score; therefore, the recorded total score ranged from 2 to 8 and can be converted to the standard 0–6 score by subtracting 2 from the total score. The scale consists of two items and uses a 4-point Likert format, with responses ranging from 1 (not at all) to 4 (nearly every day). Higher total scores indicate more severe anxiety symptoms. In the present sample, the Cronbach’s alpha coefficient was 0.852.

#### 2.2.4 Physical exercise

For the present investigation, physical exercise was assessed using the Physical Activity Rating Scale revised by Liang (47). This measure has been widely used in Chinese adolescent and student samples to assess overall physical exercise level (48). The scale includes three items assessing exercise intensity, frequency, and duration. A 5-point Likert rating system was adopted for item response scoring, with the final physical activity score computed through the predefined formula: Physical Activity Level = Exercise Intensity × (Exercise Duration - 1) × Exercise Frequency. Final total scores span a range of 0 to 100, where higher numerical values correspond to more robust levels of physical exercise engagement. Because the three items reflect different behavioral components of physical exercise rather than highly homogeneous indicators of a single latent construct, Cronbach’s alpha should be interpreted cautiously. For the sample recruited in this investigation, the Cronbach’s alpha coefficient of this instrument reached 0.653.

### 2.3 Covariates

In this study, the following variables were treated as covariates: gender, age, grade level, only-child status, hukou status (China’s household registration system, categorized as rural or urban household registration), and boarding status.

### 2.4 Statistical analysis

Data were analyzed using SPSS 26.0 and the PROCESS macro. Descriptive statistics and Pearson correlation analyses were first conducted. Mediation and moderated mediation analyses were then performed using PROCESS Models 4 and 8, respectively. Gender, age, grade level, only-child status, hukou status, and boarding status were included as covariates. Continuous variables involved in the interaction terms were standardized before the interaction terms were created. Bootstrap confidence intervals were estimated using 5,000 resamples. Effects were considered statistically significant when the 95% confidence interval did not include zero or when p < 0.05.

## 3 Results

### 3.1 Common method bias test

To assess the impact of common method bias, Harman’s single-factor test was performed. The results indicated that without factor rotation, four factors had eigenvalues greater than 1. The first factor explained 20.564% of the variance, which is below the 40% threshold. Therefore, no significant common method bias was found in this study.

### 3.2 Correlation analysis

As detailed in **Table 1**, BrainRot exhibits a significant positive correlation with anxiety (r = 0.329, p < 0.001). BrainRot was negatively related to cognitive flexibility (r = -0.286, p < 0.001) and physical exercise (r = -0.105, p < 0.001). Anxiety, in turn, shows a significant negative correlation with cognitive flexibility (r = -0.357, p < 0.001), alongside a corresponding significant negative correlation with physical exercise (r = -0.114, p < 0.001). Physical exercise was positively related to cognitive flexibility (r = 0.123, p < 0.001).

**Table 1:** Correlation analysis.

Variables	1	2	3	4
1 BrainRot	–			
2 Cognitive flexibility	-0.286***	–		
3 Anxiety symptoms	0.329***	-0.357***	–	
4 Physical exercise	-0.105***	0.123***	-0.114***	–

*p < 0.05, **p < 0.01, ***p < 0.001.

### 3.3 Mediation model testing

**Figure 2:**
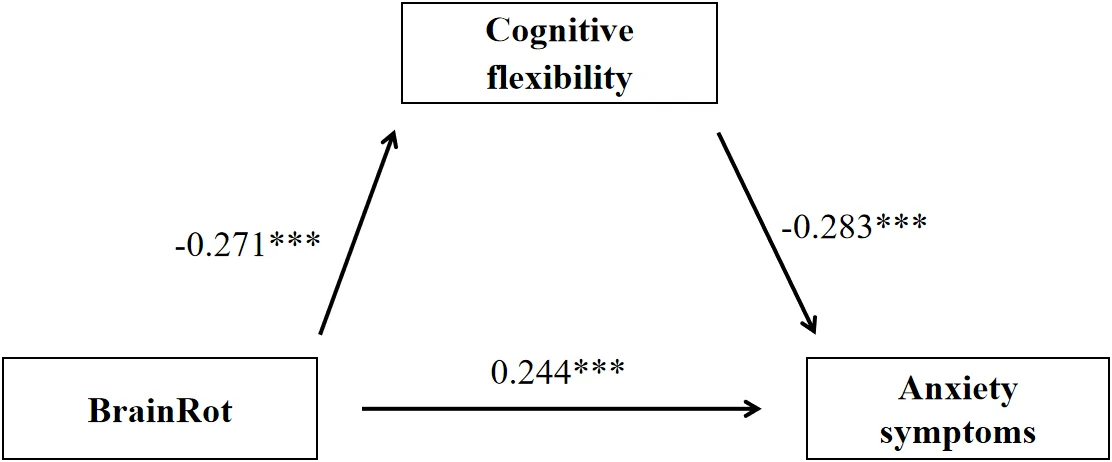
Mediation model diagram. ***p < 0.001.

After controlling for demographic variables, the findings reported in **Table 2** and **Figure 2** show that BrainRot was significantly positively related to Anxiety symptoms. This relationship remained statistically significant after the mediating variable was included in the model. In addition, BrainRot showed a significant negative association with cognitive flexibility, and cognitive flexibility was inversely related to Anxiety symptoms levels. The proportions of the path effects are presented in **Table 3**.

**Table 2:** Mediation model test.

Outcome variable	Predictor variable	β	SE	t	R²	F
Anxiety symptoms	BrainRot	0.321	0.017	19.192***	0.116	60.716***
	Gender	0.034	0.017	2.046*		
	Age	0.003	0.025	0.136		
	Grade	0.028	0.026	1.104		
	Hukou status	-0.012	0.019	-0.661		
	Only child status	0.050	0.017	2.996**		
	Living on campus	-0.049	0.018	-2.656**		
Cognitive flexibility	BrainRot	-0.271	0.017	-15.997***	0.093	47.391***
	Gender	-0.037	0.017	-2.192*		
	Age	-0.082	0.025	-3.200**		
	Grade	-0.012	0.026	-0.463		
	Hukou status	-0.003	0.019	-0.153		
	Only child status	-0.028	0.017	-1.622		
	Living on campus	0.009	0.019	0.491		
Anxiety symptoms	BrainRot	0.244	0.017	14.672***	0.188	94.395***
	Cognitive flexibility	-0.283	0.017	-17.091***		
	Gender	0.024	0.016	1.478		
	Age	-0.020	0.024	-0.816		
	Grade	0.025	0.025	1.014		
	Hukou status	-0.013	0.018	-0.735		
	Only child status	0.043	0.016	2.640**		
	Living on campus	-0.046	0.018	-2.625**		

*p < 0.05, **p < 0.01, ***p < 0.001.

**Table 3:** Mediation model path analysis.

Intermediate path	Effect size	SE	Bootstrap 95% CI	Proportion of mediating effect
Total effect	0.321	0.017	0.288, 0.353	
Direct effect	0.244	0.017	0.211, 0.276	
Total indirect effect	0.077	0.007	0.062, 0.092	23.99%

### 3.4 Moderated mediation model testing

After controlling for gender, age, grade, only-child status, hukou status, and boarding status, the moderated mediation model was examined. As shown in **Table 4**, BrainRot was negatively associated with cognitive flexibility (β = -0.267, p < 0.001) and positively associated with anxiety symptoms (β = 0.240, p < 0.001). Cognitive flexibility was negatively associated with anxiety symptoms (β = -0.281, p < 0.001). Physical exercise was positively associated with cognitive flexibility (β = 0.075, p < 0.001) and negatively associated with anxiety symptoms (β = -0.050, p < 0.01). The interaction between BrainRot and physical exercise was significantly associated with cognitive flexibility (β = -0.036, p < 0.05), indicating that physical exercise moderated the path from BrainRot to cognitive flexibility. The interaction between BrainRot and physical exercise was also significantly associated with anxiety symptoms (β = -0.032, p < 0.05), indicating that physical exercise moderated the direct path from BrainRot to anxiety symptoms. These results suggest that physical exercise moderated two paths in the final model: the path from BrainRot to cognitive flexibility and the direct path from BrainRot to anxiety symptoms.

**Table 4:** Moderated mediation model testing.

Outcome variable	Predictor variable	β	SE	t	R²	F
Cognitive flexibility	BrainRot	-0.267	0.017	-15.751***	0.100	39.972***
	Physical exercise	0.075	0.017	4.309***		
	Physical exercise * BrainRot	-0.036	0.017	-2.114*		
	Gender	-0.025	0.017	-1.500		
	Age	-0.075	0.025	-2.938**		
	Grade	-0.010	0.026	-0.378		
	Hukou status	0.004	0.019	0.195		
	Only child status	-0.018	0.017	-1.058		
	Living on campus	0.006	0.019	0.303		
Anxiety symptoms	BrainRot	0.240	0.017	14.425***	0.191	76.923***
	Cognitive flexibility	-0.281	0.017	-16.898***		
	Physical exercise	-0.050	0.016	-3.021**		
	Physical exercise * BrainRot	-0.032	0.016	-1.975*		
	Gender	0.018	0.016	1.097		
	Age	-0.021	0.024	-0.865		
	Grade	0.022	0.025	0.912		
	Hukou status	-0.016	0.018	-0.907		
	Only child status	0.039	0.016	2.379*		
	Living on campus	-0.046	0.018	-2.623**		

*p < 0.05, **p < 0.01, ***p < 0.001.

Further simple slope analysis helped interpret the significant interaction effects by comparing low, mean, and high levels of physical exercise, as shown in **Table 5**.

**Table 5:** Simple slopes by physical exercise level.

Physical exercise levels		Effect size	SE	t	Lower limit 95%CI	Upper limit 95%CI
Cognitive flexibility	Low	-0.235	0.022	-10.734***	-0.278	-0.192
	Medium	-0.267	0.017	-15.751***	-0.300	-0.233
	High	-0.302	0.024	-12.371***	-0.350	-0.254
Anxiety symptoms	Low	0.267	0.021	12.642***	0.226	0.309
	Medium	0.240	0.017	14.425***	0.207	0.273
	High	0.208	0.024	8.785***	0.162	0.255

***p<0.001.

**Figure 3:**
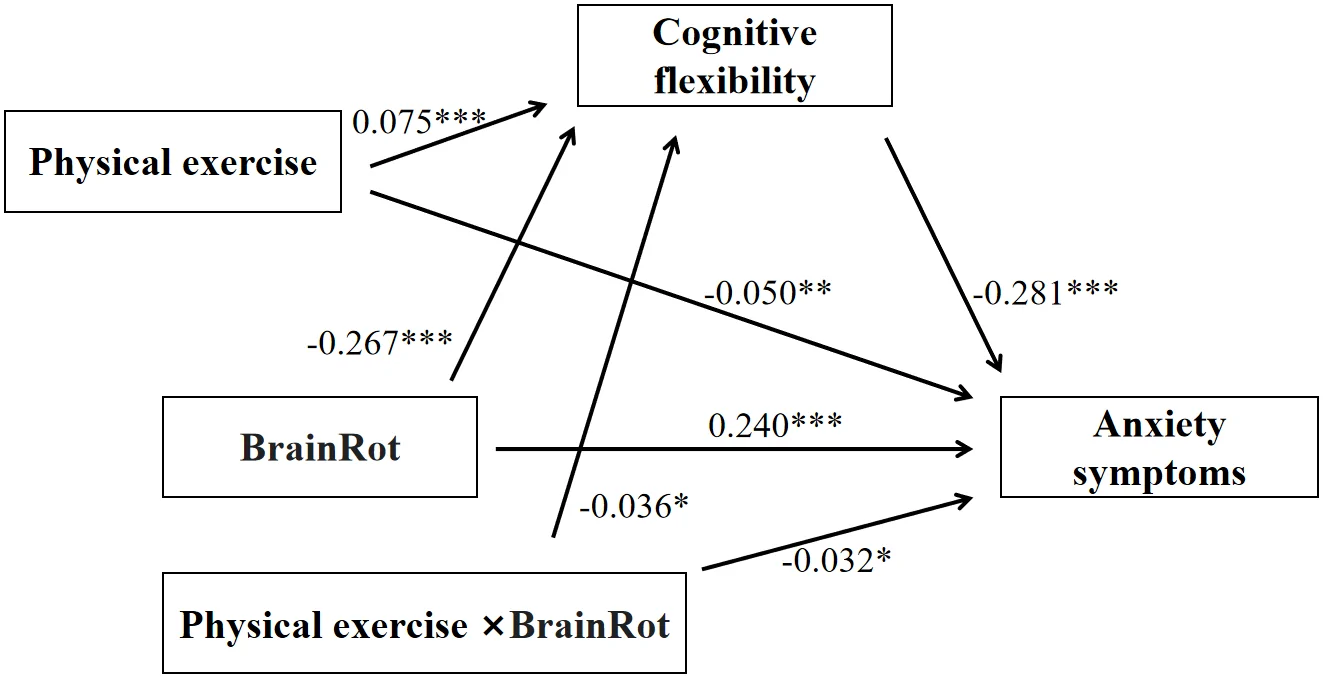
Tested moderated mediation model. *p < 0.05, **p < 0.01, *** p < 0.001.

**Figure 4:**
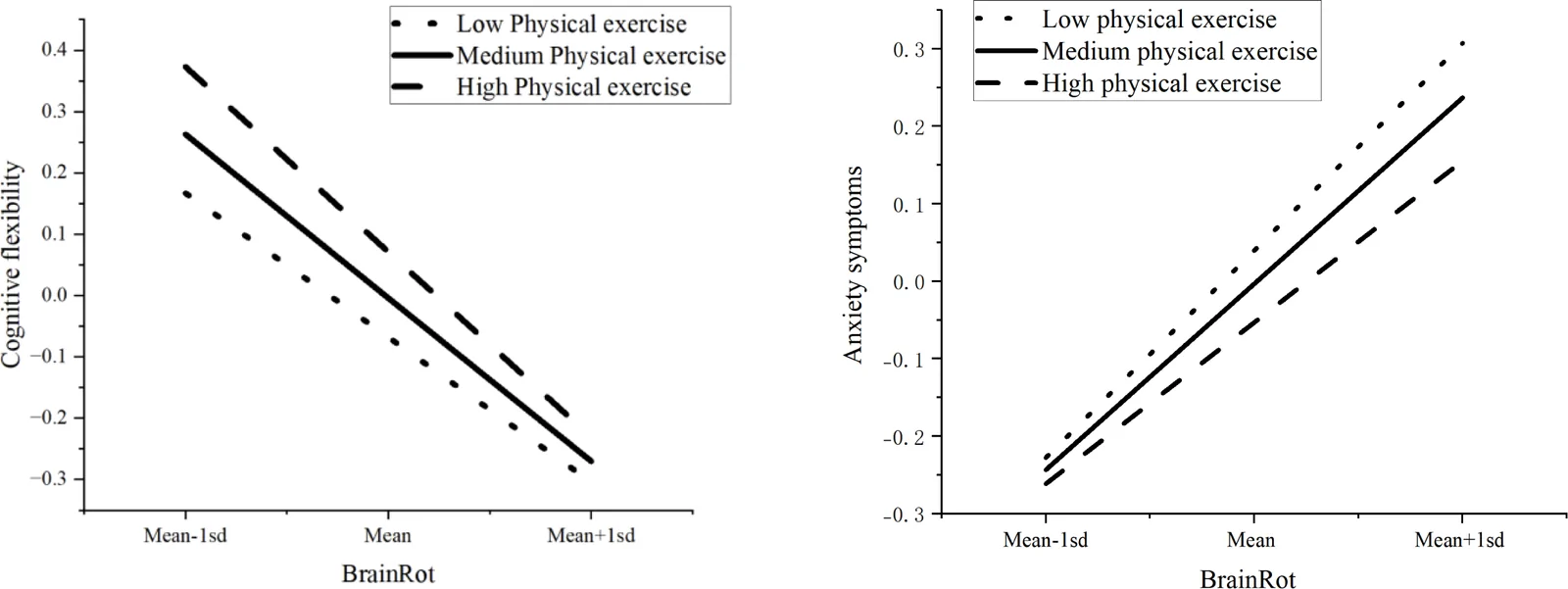
Simple slope plots.

As shown in **Table 6**, the conditional indirect effects of BrainRot on anxiety symptoms through cognitive flexibility were significant at low, mean, and high levels of physical exercise because all bootstrap 95% confidence intervals excluded zero. Specifically, the indirect effect was 0.066 at low physical exercise, 0.075 at mean physical exercise, and 0.085 at high physical exercise. However, the index of moderated mediation was not statistically significant because its bootstrap 95% confidence interval included zero, Index = 0.010, Boot SE = 0.006, 95% Boot CI [-0.002, 0.022]. Therefore, although the indirect effect was significant at different levels of physical exercise, the difference in the indirect effect across levels of physical exercise was not statistically significant. This pathway-specific pattern is further illustrated in **Figure 3**, while the corresponding simple slope patterns are shown in **Figure 4**.

**Table 6:** Conditional indirect effects of BrainRot on anxiety symptoms through cognitive flexibility at different levels of physical exercise.

Physical exercise level	Effect	Boot SE	Boot LLCI	Boot ULCI
Low	0.066	0.009	0.050	0.083
Mean	0.075	0.007	0.061	0.090
High	0.085	0.010	0.066	0.105
Index of moderated mediation	0.010	0.006	-0.002	0.022

## 4 Discussion

Using a cross-sectional design, this study examined the associations among BrainRot, physical exercise, anxiety symptoms, and cognitive flexibility among Chinese adolescents. The results showed that BrainRot was positively associated with anxiety symptoms and negatively associated with cognitive flexibility and physical exercise. Cognitive flexibility statistically mediated the association between BrainRot and anxiety symptoms. The moderation analyses further revealed a pathway-specific pattern: physical exercise weakened the positive association between BrainRot and anxiety symptoms, but it did not buffer the negative association between BrainRot and cognitive flexibility. Instead, this negative association became stronger at higher levels of physical exercise. Therefore, physical exercise should not be interpreted as uniformly protective across all pathways.

The present findings indicate that BrainRot was positively associated with adolescent anxiety symptoms, which is broadly consistent with prior evidence on problematic short-video use and mental health problems among adolescents (49). Adolescence is a critical period for the development of self-identity. For many adolescents, short-video platforms serve as significant venues for social recognition and self-worth validation. The likes, comments, and other forms of feedback on these platforms become important benchmarks for self-evaluation. The fleeting rewards and vanity in the virtual world may mask adolescents’ emotional deficits and social difficulties in real life (50). Earlier studies suggest that some adolescents may resort to excessive short-video use as a way to escape emotional distress arising from academic pressure, family conflict, or peer relationship difficulties. While this escape behavior may temporarily alleviate anxiety and stress, in the long run, adolescents lacking emotional regulation skills may become more isolated and emotionally empty, ultimately triggering anxiety (51). From a physiological perspective, the prefrontal cortex (PFC) plays a crucial role in regulating emotions, decision-making, and impulse control. Its function may be inhibited by addictive behaviors, leading to decreased emotional regulation and impulse control abilities (52, 53). Existing evidence indicates that individuals who are heavily immersed in mobile phones and social media exhibit significantly lower activity in the PFC compared to the normal population, suggesting that their self-control may be impaired. These neurophysiological findings may provide a possible background for understanding why problematic digital media use is related to emotional difficulties among adolescents (54). Thus, Hypothesis 1 was supported.

Our results further showed a negative association between BrainRot and cognitive flexibility among adolescents, aligning with previous evidence that excessive short-video use is related to poorer cognitive functioning. Short-video platforms attract users with rapid content switching, quick feedback, and continuous new information. During frequent short-video consumption, adolescents are often forced to quickly shift their attention between large amounts of information, leading to excessive depletion of the brain’s attention resources and weakening their ability to maintain sustained focus. Consistent with previous findings, cognitive flexibility not only requires individuals to switch tasks quickly but also demands that they maintain an adequate level of attention over a prolonged period. Therefore, higher BrainRot scores may be associated with poorer attention, slower information processing, and lower cognitive flexibility among adolescents. According to prior studies, short videos frequently stimulate the brain’s dopamine system through their immediate reward mechanisms, causing sustained over-secretion of dopamine. Long-term BrainRot may make adolescents dependent on frequent rewards, reducing their interest and engagement in other tasks by decreasing the brain’s reward system response (55). This preference for immediate rewards weakens adolescents’ ability to process delayed rewards, leading to poor executive function and adaptive regulation when faced with tasks that require long-term patience, planning, and cognitive flexibility adjustments. Taken together, these findings suggest that BrainRot may be associated not only with poorer cognitive functioning but also with difficulties in emotional regulation. Cognitive flexibility was negatively associated with anxiety symptoms, a finding that aligns with previous results. While the direct relationship between the two is not explicitly addressed, previous studies suggest that cognitive deficits make it difficult for individuals to regulate their emotions effectively. When adolescents face stress or negative emotions, they may struggle to adjust their emotional responses flexibly, leading to intensified emotional distress that may even evolve into anxiety symptoms (56). During their emotional and cognitive development, adolescents’ cognitive flexibility is still in the process of maturation, making them more vulnerable to emotional dysregulation (57). Adolescents with long-term deficits in cognitive flexibility are more likely to become trapped in rigid negative thinking patterns, leading to the onset and exacerbation of anxiety (58). Therefore, this study confirms the hypothesis that cognitive flexibility mediates the relationship between BrainRot and anxiety symptoms.

Contrary to the initial hypothesis, physical exercise did not show a buffering effect on the association between BrainRot and cognitive flexibility. One possible explanation is that the PARS-3 used in this study assesses overall exercise level mainly in terms of intensity, duration, and frequency, but does not distinguish between different types of physical activity, such as open-skill and closed-skill exercise. Previous research has suggested that improvements in executive function may depend more strongly on activities involving cognitive engagement, rule switching, and real-time decision-making, rather than on simple and repetitive forms of physical activity alone (59). Open-skill exercise is typically characterized by changing environments, high information-processing demands, and rapid responses, and may therefore be more beneficial for executive function than closed-skill exercise (60). Accordingly, a general index of exercise volume may not adequately capture the cognitive challenge and training quality embedded in the exercise process. Another possible explanation is that the cognitive burden associated with BrainRot may be difficult to fully offset through general physical exercise. Frequent exposure to rapidly changing and highly rewarding short-video content may continuously occupy adolescents’ attentional and executive control resources. Although physical exercise has been linked to brain health, BDNF expression, and improvements in executive function, these benefits may depend on exercise type, intensity, duration, and the quality of long-term training (61, 62). In addition, according to attention restoration theory, attentional recovery requires individuals to temporarily disengage from highly stimulating information and enter a relatively relaxing or restorative environment (63). If adolescents continue to use mobile phones, watch short videos, or engage with other digital content during exercise, the exercise experience may not truly reduce attentional load. From this perspective, higher physical exercise scores do not necessarily indicate that the exercise experience is sufficient to buffer the negative association between BrainRot and cognitive flexibility.

This study explores the interactions between BrainRot, cognitive flexibility, anxiety, and physical exercise, establishing a moderated mediation model that enriches the research frameworks of addiction psychology, emotional regulation theory, cognitive control theory, and self-determination theory. The findings help clarify the statistical pathways linking BrainRot with adolescents’ cognitive and emotional outcomes, particularly by showing that cognitive flexibility statistically mediated the association between BrainRot and anxiety symptoms. Furthermore, physical exercise plays a moderating role, indicating that physical exercise showed different moderating patterns across pathways. It weakened the positive association between BrainRot and anxiety symptoms, but it did not buffer the negative association between BrainRot and cognitive flexibility. Therefore, the role of physical exercise should be interpreted as pathway-specific rather than uniformly protective. This finding provides a useful perspective for future theoretical research, highlighting the application value of interdisciplinary theoretical models in addressing complex psychological issues. BrainRot is a phenomenon prevalent globally; however, the patterns of short-video use and their psychological correlates may vary across different cultural contexts and social environments. Future research could conduct cross-cultural comparisons to examine the associations between BrainRot, cognition, and emotions in different cultural contexts, as well as the moderating role of physical exercise. This study highlights the potential role of physical exercise in adolescent mental health promotion, particularly in relation to anxiety symptoms and emotional regulation among adolescents with higher levels of BrainRot. For adolescents, physical exercise may represent a potentially beneficial behavioral correlate of mental health and may serve as a possible intervention target for future longitudinal and experimental studies. However, the present cross-sectional findings should not be interpreted as evidence that physical exercise directly reduces BrainRot or anxiety symptoms.

This study offers a comprehensive analysis of the interactions between BrainRot, cognitive flexibility, anxiety, and physical exercise, testing a moderated mediation model that describes how these factors are statistically associated with adolescents’ mental health. Such a multidimensional analysis provides a more comprehensive perspective for both theory and practice. In terms of theoretical implications, this study may provide preliminary evidence for considering physical exercise as a behavioral correlate and a potential target for future prevention-oriented research. It suggests that physical exercise may be considered a potentially beneficial behavioral factor in relation to BrainRot and anxiety symptoms, with practical implications for adolescent mental health promotion in schools, families, and communities. Although this study used a cross-sectional design and revealed associations among variables, causal relationships cannot be inferred. Therefore, the observed mediation and moderation results should be interpreted as statistical associations rather than causal or temporal mechanisms. Future longitudinal and experimental studies are needed to clarify the temporal order and potential causal relationships among problematic short-video use, cognitive flexibility, anxiety symptoms, and physical exercise. Another limitation concerns the measurement of physical exercise. The Physical Activity Level Scale used in this study assessed only the general level of physical exercise based on intensity, duration, and frequency, but it did not provide detailed information on the specific types of sports activities, exercise contexts, exact exercise duration, participation motivation, or whether the activity was individual- or team-based. Therefore, the present study could not determine whether different forms of physical exercise had distinct associations with cognitive flexibility and anxiety symptoms. Future studies should use more detailed physical activity measures or objective monitoring tools, such as accelerometers, pedometers, or wearable devices, to capture the type, duration, context, and motivation of sports participation. Moreover, since self-report questionnaires were used in this study, individual reporting bias (e.g., adolescents underestimating or overestimating their short video use or anxiety symptoms) may affect the accuracy of the results. To improve accuracy, future research could employ multi-method data collection (e.g., behavioral observations, parent reports) to further validate the findings.

## 5 Conclusion

This study explored the relationships between BrainRot, cognitive flexibility, anxiety symptoms, and physical exercise, and developed a moderated mediation model. The results indicate that BrainRot is significantly correlated with anxiety symptoms in adolescents, and cognitive flexibility plays a mediating role in the relationship between BrainRot and anxiety. Further analysis showed that physical exercise significantly moderated the path from BrainRot to cognitive flexibility and the direct path from BrainRot to anxiety symptoms. The conditional indirect effects through cognitive flexibility were evident across low, mean, and high levels of physical exercise. These findings suggest that physical exercise played a pathway-specific moderating role, particularly for the paths from BrainRot to cognitive flexibility and anxiety symptoms. The findings suggest that higher BrainRot scores were linked to lower cognitive flexibility and higher anxiety symptoms among adolescents. Physical exercise may represent a potentially beneficial behavioral factor for adolescents with higher levels of BrainRot, particularly in relation to anxiety symptoms and emotional regulation. However, its role in the BrainRot–cognitive flexibility association was not consistent with a buffering effect and should therefore be understood as pathway-specific rather than uniformly protective.

## Data Availability

The raw data supporting the conclusions of this article will be made available by the authors, without undue reservation.
